# Intraindividual Variability across Neuropsychological Tests: Dispersion and Disengaged Lifestyle Increase Risk for Alzheimer’s Disease

**DOI:** 10.3390/jintelligence6010012

**Published:** 2018-03-01

**Authors:** Drew W. R. Halliday, Robert S. Stawski, Eric S. Cerino, Correne A. DeCarlo, Karl Grewal, Stuart W. S. MacDonald

**Affiliations:** 1 Department of Psychology, University of Victoria, Victoria, BC V8P 5C2, Canada; decarloc@uvic.ca (C.A.D.); kgrewal@uvic.ca (K.G.); smacd@uvic.ca (S.W.S.M.); 2 Institute on Aging and Lifelong Health, University of Victoria, Victoria, BC V8P 5C2, Canada; 3 School of Social and Behavioral Health Sciences, Oregon State University, Corvallis, OR 97331, USA; Robert.Stawski@oregonstate.edu (R.S.S.); cerinoe@oregonstate.edu (E.S.C.)

**Keywords:** intraindividual variability, dispersion, cognitive impairment, mild cognitive impairment, Alzheimer’s Disease, neuropsychological assessment

## Abstract

*Objective*: Increased intraindividual variability (IIV) in function has been linked to various age-related outcomes including cognitive decline and dementia. Most studies have operationalized IIV as fluctuations across trials (e.g., response latencies) for a single task, with comparatively few studies examining variability across multiple tasks for a given individual. In the present study, we derive a multivariable operationalization of dispersion across a broad profile of neuropsychological measures and use this index along with degree of engaged lifestyle to predict risk of cognitive impairment. *Participants and Methods*: Participants (*n* = 60) were community-dwelling older adults aged 65+ years (M = 74.1, SD = 6.5) participating in a cross-sectional investigation of risk factors for amnestic mild cognitive impairment (a-MCI) and probable Alzheimer’s Disease (AD). Participants were classified into three subgroups based on test performance and clinical judgement. Healthy controls (*n* = 30) scored better than −1 SD relative to existing norms on all classification measures, in the absence of memory complaints or functional impairments. The a-MCI group (*n* = 23) had self- or informant-reported memory complaints and scored 1 SD or more below the mean for at least one memory task while scoring better than 1 SD below the mean for all other cognitive domains, in the absence of functional impairments. The AD group (*n* = 7) scored at least 2 SD below the mean for two cognitive domains (including memory) with impairments in functioning. Measures spanned a range of cognitive domains (episodic memory, executive function, language), with the derived dispersion estimates reflecting variability across an individual’s neuropsychological profile relative to the group average. Further, an Activities Lifestyle Questionnaire, indexing social, cognitive, and physical behaviors, was administered to assess the protective benefits of engaged lifestyle. *Results*: Multinomial logistic regression models examined the risk of being classified as a-MCI or AD as a function of increased dispersion, (dis)engaged lifestyle, and their interaction. Greater dispersion was associated with an increased likelihood of being classified with AD, with protective engaged-lifestyle benefits apparent for a-MCI individuals only. *Conclusion*: As a measure of IIV, dispersion across neuropsychological profiles holds promise for the detection of cognitive impairment.

## 1. Introduction

Intraindividual variability (IIV) is increasingly employed as a metric of functioning across behavioral (e.g., response time) [1], physical (e.g., gait) [2], physiological (e.g., heart rate) [3], and neurophysiological (e.g., blood-oxygen-level-dependent signal) functioning [4]. Research has shown that IIV often confers information that is independent to that of central tendency metrics and that in some cases, IIV is more sensitive to deleterious health outcomes and pathophysiological processes [5,6]. Most commonly, IIV refers to inconsistency in function (e.g., behavioral performance) within-persons and across time, and can be indexed across broader (e.g., week-to-week) or narrower (e.g., trial-to-trial performance) time scales. Greater IIV in trial-to-trial behavioral performance has been associated with risk for decline in cognitive status, including Mild Cognitive Impairment and dementia [7,8,9]. Evidence for the mechanisms driving increased behavioral inconsistency has pointed to compromised neural integrity at anatomical, functional, neuromodulatory and genetic levels [10], further implicating the potential utility of IIV for detecting early cognitive decline.

Although inconsistency in behavioral performance (i.e., IIV over time) has elucidated several insights in terms of late-life developmental and health-related outcomes, less is understood about IIV in terms of performance across different tasks within individuals. Dispersion refers to IIV across multiple different indicators within-persons; most typically, across cognitive and neuropsychological tasks [11,12] and may reflect similar underlying processes (e.g., age related changes in neurological integrity) to those identified for inconsistency [12]. Like inconsistency, dispersion is sensitive to age differences in late-life, with old-old adults (75–92 years) demonstrating higher levels of dispersion relative to young-old adults (65–74 years) [13]. Differences in dispersion have also been observed across broader segments of the lifespan. A recent investigation demonstrated that dispersion across working memory and RT tasks may reflect different developmental phenomena, with greater dispersion in RT tasks observed during childhood and older adulthood and greater dispersion in working memory observed during young adulthood [14].

In addition to developmental phenomena, dispersion has been examined in the context of acquired and neurodegenerative conditions impacting cognitive performance. While some studies have found distinct and meaningful profiles of dispersion [13,15], others have focused on the relative magnitude of overall dispersion between groups with cross sectional [14,16] and longitudinal designs [11,17,18]. Rabinowitz and Arnett [16] found that greater dispersion was associated with post-concussive cognitive dysfunction in a sample of college athletes across a battery of computerized and paper-pencil neuropsychological tasks, suggesting that such disparate profiles across a broad range of tasks may be sensitive to mild neurological trauma. Similarly, greater dispersion in neuropsychological test performance predicted incident dementia, independent of performance on each individual test, in a population-based longitudinal study of older adults [17]. This finding was replicated in a larger sample of older women, such that greater baseline dispersion subsequently predicted probable dementia; however, the effect was attenuated in individuals with higher verbal episodic memory scores [18]. Previous investigations of conversion from cognitively-impaired-not-demented to dementia status using cluster analyses also suggest that memory and verbal dysfunction are most predictive of conversion to dementia [15]. Independent of more nuanced cognitive profiles, greater dispersion has also been associated with poorer activities of daily living (ADLs) in older adults [11]. In this study, dispersion was not associated with age, level of education or lifestyle activity levels. Notably, however, lifestyle activity levels were coarsely indexed, with only a marginal distinction observed between different types of activities (e.g., social, physical, cognitive) and for a relatively restricted response range. 

Lifestyle, including engagement in cognitive, physical, and social activities, plays a critical role in psychosocial well-being and maintaining neurological integrity [19]. For example, higher lifetime cognitive activity and current level of physical activity in older adults is associated with the presence of fewer white matter lesions, which are in turn associated with greater neural integrity and global cognitive functioning [20]. Cognitive reserve-enhancing factors, including late-life engagement in cognitive, physical, and social activity, were recently demonstrated to reduce the relative risk of dementia in older adults [21]. In older adulthood, engagement in protective lifestyle activities may both contribute to and be facilitated by healthy cognitive functioning [22]. Recent longitudinal evidence using latent growth curve modelling suggests that engagement in cognitive, social, and physical activity is associated with less cognitive decline in late-life [23], affirming a long line of assertions implicating engaged lifestyle as a target for preventive efforts. 

As markers of cognitive decline and dementia risk, an index of dispersion across a comprehensive battery of cognitive performance measures as well as a psychometrically well-validated measure of lifestyle activities have yet to be thoroughly examined both individually and simultaneously. The present study sought to examine two primary research questions. First, can previous findings linking dispersion to cognitive subgroup differences be replicated and extended to demonstrate the sensitivity of dispersion across a broad neuropsychological-assessment profile to amnestic-MCI (a-MCI) and probable Alzheimer’s disease (AD)? Although dispersion has been regularly observed in relatively impaired individuals, the sensitivity of dispersion for predicting a-MCI or for AD (in contrast to all-cause dementia) is less clear. Second, does living an engaged lifestyle, characterized by relatively high frequency of participation in social, physical, and cognitive activities and indexed employing a psychometrically well-validated measure, confer protective benefits independent of neuropsychological dispersion? Given the association between cognitive status and engaged lifestyle, engagement in activity may serve as an avenue to decrease cognitive dispersion and promote greater well-being overall.

## 2. Method

### 2.1. Participants

Participants were community-dwelling older adults from Victoria, BC, Canada participating in The PREVENT Study; a cross-sectional multi-factorial (e.g., biological, physiological, environmental) investigation of risk factors for a-MCI and probable AD. Participants were recruited through descriptions of the study in various news outlets and presentations to community groups; individuals aged 65 years and older were sought in an effort to target late-onset pathology. Exclusionary criteria for participation focused on factors that could directly result in cognitive deficits or impairment not reflective of emerging neurodegenerative conditions consistent with AD or its prodrome. These included (a) newly diagnosed psychiatric disturbance within the past year (i.e., Major Depressive Disorder); (b) history of a chronic neurological condition (i.e., Parkinson’s disease, brain tumor); (c) episode(s) of cardio- and/or cerebro-vascular disease (i.e., heart attack, stroke, heart surgery) within the past year; and (d) other factors that could contribute to changes in cognitive functioning (i.e., head injury, vitamin deficiency). Severe sensory and/or motor impairment (i.e., unable to read newspaper-sized print with glasses, difficulty writing or pressing keys on a keyboard, or unable to hear a normal spoken conversation adequately with the use of a hearing aid) were also used as exclusionary criteria, given the nature of participation.

### 2.2. Cognitive Status Classification

Participants were classified as either healthy control (HC, *n* = 30), a-MCI (*n* = 23) or AD (*n* = 7), based on a standard and objective classification system involving both neuropsychological test scores (based on 7 classification measures yielding 8 different scores) and clinical judgement. To meet criteria for the HC group, participants were required to (a) score better than 1.0 SD below the mean for all cognitive domains, and (b) report no subjective memory complaints or impairment in social, occupational, or daily functioning during interview. To meet criteria for the a-MCI group [24,25,26], participants were required to (a) score at least 1.0 SD below the mean in the memory domain; (b) score better than 1.0 SD below the mean in all other cognitive domains; (c) report at least one subjective complaint associated with memory during interview; and (d) report an absence of impairment in social, occupational or daily functioning during interview. To meet criteria for the probable AD group, consistent with DSM-IV-TR guidelines [27], participants were required to (a) score at least 2.0 SD below the mean for memory and in one other cognitive domain; (b) have subjective or collateral-reported significant declines from previous levels of functioning in both domains that were gradual and progressive (i.e., versus acute declines that are more likely to be associated with cerebrovascular events or other pathophysiological changes not associated with AD), with (c) these deficits resulting in impairments in social, occupational and/or daily life functioning. These latter two criteria were assessed during interview. Table 1 depicts select demographic characteristics for each group.

There were no significant differences between-groups in terms of chronological age (*F*(2,57) = 0.807, *p* = 0.45), or years of education (*F*(2,57) = 1.164, *p* = 0.32). Significant between-group differences were found in terms of self-reported memory function in the 30 days prior to the screening interview (*F*(2,54) = 5.757, *p* < 0.005, η^2^ = 0.18), with the HC group (*m* = 7.41, *SD* = 1.28) reporting greater memory function relative to the a-MCI (*m* = 5.81, *SD* = 2.13) group. No differences were observed between the a-MCI and AD (*m* = 6.00, *SD* = 2.24) groups, or between the AD and HC groups (based on post-hoc comparison using Tukey’s HSD)^1^.

## 3. Measures

### 3.1. Test Battery

The test battery included measures spanning the following cognitive domains, as outlined in Table 2; global cognitive functioning (Modified Mini-Mental State Test (3MS)), auditory attention (WAIS-R Digit Span (Total score)), auditory working memory (WAIS-R Digit Span Backwards), visual memory (Benton Visual Retention Task-BVRT), auditory immediate and delayed memory (Rey Auditory Verbal Learning Task (RAVLT; A1-5 Total, A6 (short delay interference), A7 (long delay), d’ (recognition)), executive functioning (WAIS-R Similarities, Trail Making Test B-TMT-B, Mental Alternation Test-MAT), language (Controlled Oral Word Associations Test-COWAT, Animal Naming, North American Adult Reading Test-NAART), visuospatial ability (WAIS-R Block Design), and processing speed (Trail Making Test A-TMT-A, WAIS-R Digit Symbol, Serial Response Time-SRT, Lexical Decision Task (accuracy and RT)). Normative data from the Canadian Study of Health and Aging (CSHA) were used to derive T-scores for the WAIS-R short-form subtests, RAVLT interference (A6) and long-delay (A7), BVRT, COWAT (using CFL) and Animal Naming [28]. Normative data from the Mayo’s Older Americans Normative Studies (MOANS) were used to derive T-scores for TMT-A, TMT-B [29], and the immediate recall trials of RAVLT (A1-5) [30], due to the lack of available normative data for these tests in the CSHA study. Individuals over the age of 90 (*n* = 1; age 93, a-MCI group) were compared to 90-year-olds in the CSHA reference sample. In addition to the neuropsychological tests administered, a structured interview with the participant and/or their family member was conducted to obtain self-report or collateral-report information pertaining to the participant’s social, occupational, or daily life functioning. During the screening interview, participants were asked to rate their level of memory functioning on a scale of 1–10 (1 = worst, 10 = best) over the past 30 days. As noted in the table and as described further in Section 4.1, select measures were reserved for classification purposes solely, with additional independent measures employed for deriving estimates of dispersion. Group comparisons across each of the cognitive measures is available in the Supplementary Materials online.

### 3.2. Lifestyle Activities

The revised Activity Lifestyle Questionnaire (ALQ) [31], a self-report activity questionnaire of adult leisure activities, was initially developed and administered for the Victoria Longitudinal Study (VLS) [32]. The revised version of the VLS-ALQ employed in this study enhanced the content validity of the scale by including supplemental items on physical and social activities. The structure of this revised ALQ was validated using confirmatory factor analyses in independent samples. Good psychometric properties (reliability, convergent and discriminant validity) for the ALQ support the use of its subscales as indicators of leisure activities across the lifespan [31]. For each of the items, individuals self-reported the frequency of participation for a given activity within the past year on a 9-point scale (0 = never, 1 = less than once a year, 2 = about once a year, 3 = 2 or 3 times a year, 4 = about once a month, 5 = 2 or 3 times a month, 6 = about once a week, 7 = 2 or 3 times a week, 8 = daily). An aggregate score of *lifestyle engagement* was computed as the total score summing across each of the subscales. The confirmatory factor validation of the revised ALQ [31] yielded a well-fitting higher-order general activity factor in two independent samples, thereby supporting the use of a single lifestyle engagement score in the present investigation. The 11 first-order activity factors approximate social, physical, and cognitive pursuits, briefly summarized in the following sections.

### 3.3. Physical Activities

The physical activities included in the lifestyle engagement aggregate score were derived from a subset of 10 items from the revised VLS-ALQ. These 10 individual items indexed various physical activities including select exercises (e.g., swimming, cycling), outdoor activities (e.g., sailing, fishing), sports (e.g., tennis, bowling, golf), aerobics (e.g., cardiovascular workouts), flexibility training (e.g., yoga, tai chi), walking, dancing, and resistance training (e.g., weight lifting, strength training).

### 3.4. Social Activities

Similarly, the social activities in the lifestyle engagement score were based upon a subset of 15 items from the revised VLS-ALQ. These 15 items indexed various socially-engaging activities including visiting friends/relatives, dining out at restaurants, hosting dinner parties, attending church, attending club meetings, volunteering, as well as attending public events or lectures.

### 3.5. Cognitive Activities

Cognitive activities in the aggregate score were based upon a subset of 27 items from the revised VLS-ALQ. These individual items reflected leisure activities that are cognitively stimulating such as playing a musical instrument, photography, computer use, tax preparation, engaging in business activity, reconciling a financial statement, mathematical calculations (with and without a calculator), creative writing, reading, taking continuing education courses, studying a second language, crosswords and playing games (e.g., chess, checkers, knowledge games, word games, jigsaw puzzles).

## 4. Results

### 4.1. Dispersion Index

**Figure 1:**
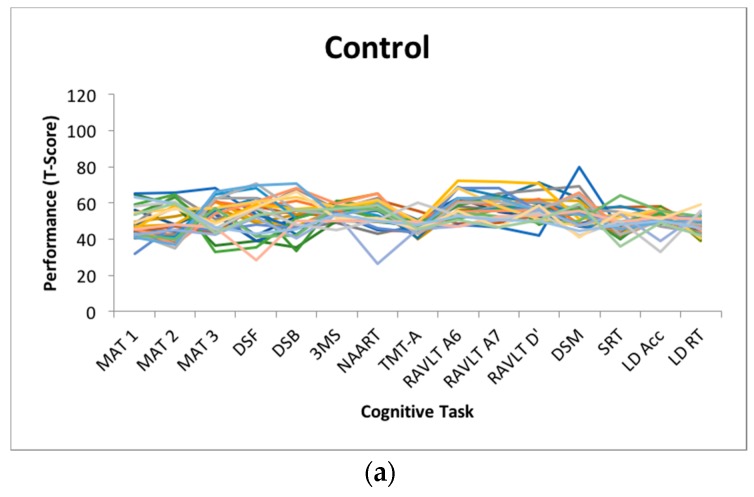

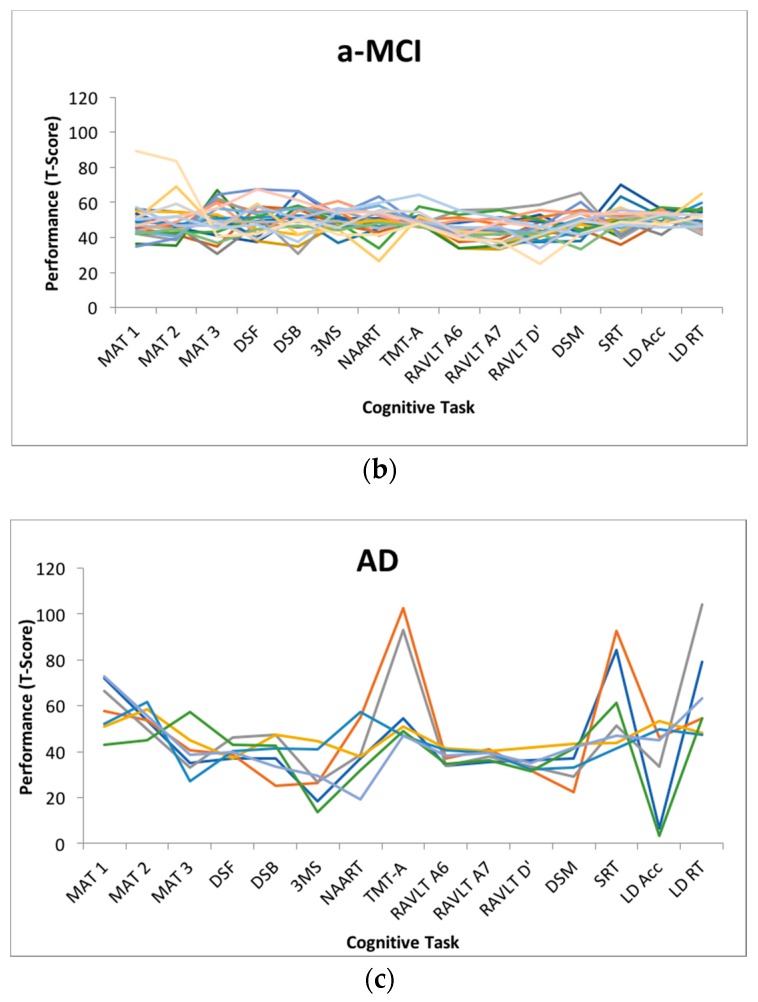
Dispersion profiles for each group of participants, classified as (**a**) healthy control (HC), amnestic (**b**) Mild Cognitive Impairment (a-MCI) and (**c**) Alzheimer’s disease (AD).

Dispersion is a measure of intraindividual variability that is computed as an intraindividual standard deviation (ISD), reflecting performance fluctuations across a profile of cognitive measures within an individual. Dispersion profiles were derived using a regression technique, which computes ISD scores from standardized test scores [11,12]. Test scores of interest (MAT, Digit Span Forward and Backward, 3MS, NAART, TMT-A, RAVLT A6, A7 and recognition, Digit Symbol, SRT, Lexical Decision) were initially regressed on linear and quadratic age trends to control for group differences in mean performance, given that greater variance tends to be associated with greater means [33,34] and that mean-level performance is likely to differ across age bands present in the current sample. The resulting residuals from these models were standardized as T-scores (M = 50, SD = 10), with ISDs subsequently computed across these residualized test scores. The resulting dispersion estimate, indexed on a common metric, reflects the amount of variability across an individual’s neuropsychological profile relative to the group average level of performance; higher values reflect greater IIV in cognitive function. Dispersion was computed across all test scores in the battery that were not used for cognitive classification (*n* = 15). Across the entire sample, the average dispersion score was 8.69 (*SD* = 4.15) T-score units. Figure 1 depicts the magnitude of dispersion within each cognitive status subgroup.

### 4.2. Between-Group Differences in Dispersion and Lifestyle

Employing analysis of variance, between-group differences were observed on average amount of dispersion (*F*(2,57) = 25.326, *p* < 0.001, η^2^ = 0.47), with the AD group (*m* = 16.42, *SD* = 7.26) scoring higher than the a-MCI (*m* = 7.39, *SD* = 2.69) and HC (*m* = 7.87, *SD* = 1.45) groups, who did not differ based on post-hoc comparisons using Tukey’s HSD. Between-group differences were also observed in terms of overall engaged lifestyle summary score, based on the ALQ (*F*(2, 56) = 7.564, *p* < 0.001, η^2^ = 0.21), with post hoc comparisons indicating that the HC group (*m* = 154.86, *SD* = 28.35) reported more engagement relative to the AD group (*m* = 102.71, *SD* = 52.72), but not the a-MCI group (*m* = 132.91, *SD* = 33.60).

### 4.3. Risk of Cognitive Impairment

Multinomial logistic regression models were used to examine the likelihood of being classified as a-MCI or AD, relative to HC, using dispersion, lifestyle engagement (total ALQ score) as well as demographic covariates (age and education) as predictors. Independent of age and education, increased dispersion was associated with a greater likelihood of being classified as AD (OR = 1.20, CI = 1.04, 1.38, *p* < 0.05), χ^2^(6) = 24.223 *p* < 0.001, Nagelkerke’s R-squared = 0.39. For every T-score unit increase in dispersion (approximately 1/10 of a standard deviation), the likelihood of being classified as AD increased by 20%. Dispersion was not, however, associated with a greater likelihood of being classified as a-MCI. Similarly, a more engaged lifestyle was associated with a reduced likelihood of being classified as either a-MCI (OR = 0.92, CI = 0.85, 0.99, *p* < 0.05) or AD (OR = 0.84, CI = 0.74, 0.94, *p* < 0.005), independent of age and education, χ^2^(6) = 18.454 *p* < 0.005, Nagelkerke’s R-squared = 0.31. For every T-score unit increase in the engaged-lifestyle score, the likelihood of being classified as a-MCI or AD was reduced by 8% and 16, respectively.

With both the dispersion and engaged lifestyle scores entered simultaneously in a multinomial logistic regression model, engaged lifestyle remained protective against a-MCI (OR = 0.90, CI = 0.83, 0.98, *p* < 0.05), but not AD (OR = 0.88, CI = 0.73, 1.05, *p* > 0.05), χ^2^(8) = 32.508 *p* < 0.001, Nagelkerke’s R-squared = 0.50. Conversely, dispersion remained predictive of AD (OR = 1.23, CI = 1.02, 1.47, *p* < 0.05), but not a-MCI risk (OR = 0.97, CI = 0.85, 1.10, *p* > 0.05). Independently, cognitive dispersion was predictive of cognitive impairment for more substantial (AD) degrees of impairment only, while lifestyle engagement was predictive of cognitive impairment risk for moderate (a-MCI) and substantial (AD) impairment; however, when examined simultaneously, lifestyle engagement was sensitive only to moderate impairment (a-MCI), while dispersion was sensitive only to the most impaired cognitive status (AD). Lastly, we computed a model specifying both main effects (dispersion and total ALQ) as well as the interaction between dispersion and total ALQ to evaluate the potential modulating influence of engaged lifestyle on the neuropsychological dispersion-cognitive impairment association. No significant dispersion-engaged lifestyle interactions were observed (*p* > 0.05) for risk of either a-MCI or AD, χ^2^(10) = 33.190 *p* < 0.001, Nagelkerke’s R-squared = 0.50.

## 5. Discussion

As a measure of intraindividual variability that is sensitive to developmental phenomena and to deleterious health outcomes in late-life, dispersion (i.e., intraindividual variability across a profile of tests) has received less attention relative to the more commonly employed measure of inconsistency (i.e., intraindividual variability in performance across time). Like inconsistency, dispersion has shown sensitivity to acquired [16] and neurodegenerative conditions [9,11,13,15,17,18], including MCI and dementia classification. In this context, greater dispersion observed for individuals not yet presenting with additional symptomatology (e.g., functional impairment, subjective memory complaints) may stem from early declines in neural integrity reflective of the dementia prodrome (e.g., medial temporal lobe atrophy). Further, engagement in lifestyle activities play a known protective factor in late-life [19,20,21,22,23] and is important for maintaining healthy cognitive functioning. Although previous investigations of dispersion have found that greater dispersion was related to poorer ADLs, but not to overall activity levels [11], the relationship between activity levels and dispersion in late-life has not been examined using psychometrically-validated measures of lifestyle activities in a sample of rigorously classified older adults.

The present study sought to replicate previous findings linking dispersion to cognitive subgroups and to examine whether lifestyle activity was protective against risk for cognitive decline, given recent findings demonstrating the sensitivity of engaged lifestyle in predicting dementia risk [21]. Relative to previous investigations examining dispersion-cognitive impairment links, a particular strength of the present study concerns the rigor of the screening criteria for indexing AD. We observed group differences in dispersion, computed across a battery of 15 cognitive and neuropsychological tests, such that the AD group showed greater dispersion relative to the HC group and those classified as a-MCI. Considerable variance in dispersion was also observed within the HC group. Greater dispersion emerged as a significant predictor in examining the risk of AD classification relative to HC. Interestingly, dispersion did not emerge as a significant predictor of a-MCI classification, relative to HC. Among the potential reasons, this finding may be due to the well-known heterogeneity between-individuals for even the most rigorously-screened MCI groups [35]. The lack of differentiation may also be due to the nature of the tasks included in the broad profile dispersion computation. Given the nature of a-MCI and the circumscribed memory impairments that represent a hallmark of the condition, a-MCI individuals may only demonstrate greater dispersion with the inclusion of sufficient short-term and episodic memory measures in the battery. As the condition progresses towards AD pathology, inconsistent cognitive performance in domains that are initially more robust may become more apparent. As most of the memory tests included in the present battery were used for cognitive classification, they were necessarily excluded from the dispersion computation.

In addition to demonstrating lower levels of dispersion, the HC group reported greater engagement in physical, cognitive, and social lifestyle activities on a comprehensive and psychometrically-validated measure of adult lifestyle activities [31]. Independent of the effects of dispersion, engaged lifestyle was protective against a-MCI, but not AD classification. This finding is consistent with the documented importance of an engaged lifestyle for maintaining cognitive function and mitigating cognitive impairment [19,23]. As central nervous system (CNS) impairment becomes more progressive and pronounced, engagement in lifestyle activities may no longer be as protective against cognitive impairment. This may be especially the case for well-characterized AD individuals who are also demonstrating greater inconsistency across cognitive areas. Further, our findings are consistent with claims that dispersion, as a marker of CNS integrity, may be particularly sensitive for detecting individuals with progressive neuropathology [12,17]. Notably, regarding detection of AD risk in particular, dispersion (i.e., inconsistency across tasks) can be computed using both speed and accuracy measures, which is important as some of the most extensively researched and validated standardized measures used in clinical practice to assess neuropsychological functioning yield accuracy scores only. As researchers attempt to better understand the relationship between enrichment effects on cognitive development, including lifestyle engagement, such validated measures that predict success in more complex day-to-day behaviors may afford greater ecological validity. This is especially the case as intervention efforts shift away from cognitive training in isolation to cognitive training in a more applied context to facilitate greater far transfer and generalization of the intervention [19].

### Limitations and Future Directions

Several limitations and future directions are noted. As is common for clinical neuropsychology studies, the present study contained only a small sample of individuals diagnosed as probable-AD, which limited statistical power and precluded an examination of more nuanced associations between dispersion and lifestyle activities that share theoretical underpinnings (e.g., executive functioning and engagement in cognitively demanding lifestyle activities). Future studies may consider examining the association between dispersion within a particular cognitive domain and more specific lifestyle activities (e.g., a physically-engaged lifestyle) to help further elucidate the potential utility of dispersion to inform intervention strategies (e.g., to target an area of lifestyle activity that draws upon cognitive processes showing early decline). Contrasting specific profiles of dispersion may also be useful in determining which cognitive domains show greater and lesser variability within-persons of a given cognitive status [13,15], given that isolated impairments in some clinical populations will result in fairly stable scores within a domain (e.g., consistently low memory performance in an AD sample). Further, examining the comparative utility of different operationalizations of IIV (e.g., dispersion and response time inconsistency) remains an important avenue for future research. Future investigations employing dispersion should be mindful of how the nature of the tasks selected for the computation will affect results. For example, across a broader profile of tasks spanning crystallized to fluid abilities, we might expect greater dispersion profiles (intact performance on some measures, impaired on others) for the cognitively impaired group vs. controls. Examining the comparative protective benefit across subtypes of lifestyle engagement also remains an important topic for further investigation.

Given the comparatively greater number of empirical studies examining IIV across trials (e.g., inconsistency) in other areas of functioning (e.g., heart rate, neural activity, gait), future dispersion studies may also consider examining dispersion across multiple domains of functioning, especially to the extent that increased dispersion may be driven by common underlying systems. Motoric Cognitive Risk Syndrome (MCR) is characterized by cognitive and gait dysfunction and is both highly prevalent in older adults [36] and sensitive to risk for dementia [37]. MCR represents an opportunity for future investigation of multi-domain dispersion (i.e., gait and cognition) that may yield useful insights into the etiology of the condition and the potential predictive utility of dispersion, beyond single-domain dispersion in isolation. 

## 6. Conclusions

The results of this study replicate previous findings suggesting that dispersion across cognitive tests is sensitive to cognitive status in late-life, particularly when individuals are relatively impaired. Individuals who are disengaged from cognitive, physical, and social lifestyle activities are more likely to be classified as having a-MCI as the stability of their cognitive processes decreases, relative to those who are more engaged. Notably, this relationship may be viewed both ways; that is, disengaged lifestyle may lead to atrophy of cognitive and neurological systems that may otherwise be stimulated through activity engagement. Conversely, cognitive impairment may preclude engagement in certain activities that rely on cognitive processes that have become compromised. Regardless, individuals at risk may be better identified through an assessment of both dispersion and lifestyle activities. Further, interventions for those at risk may consider targeting activity engagement, while monitoring cognitive dispersion as a marker of stability and risk for deleterious health outcomes.

## Figures and Tables

**Table 1:** Demographic characteristics by group.

Characteristics	HC	a-MCI	AD
*n*	30	23	7
Sex	23 females; 7 males	9 females; 14 males	3 females; 4 males
Age	73.57 (6.40)	73.95 (6.79)	77.00 (5.68)
Years of Education	15.05 (2.56)	15.39 (3.97)	13.29 (2.98)
Self-reported memory	7.41 (1.28)	5.81 (2.13)	6.00 (2.24)

**Table 2:** List of Neuropsychological and Cognitive tasks.

Cognitive Domain	Test	Scores
Global Cognition	3MS	Total
Attention	WAIS-R Digit Span Forwards	Total
Working Memory	WAIS-R Digit Span Backwards	Total
Memory	Benton Visual Retention Task (BVRT) Rey Auditory Verbal Learning Task (RAVLT)	BVRT—Total * RAVLT—A1-5 * total, A6, A7, d’
Executive Function	WAIS-R Similarities Trail Making Test B (TMT-B) Mental Alternation Test (MAT)	Similarities—Total * TMT-B—Total * MAT—1, 2, 3 totals
Visuo-construction	WAIS-R Block Design	Total *
Language	Controlled Oral Word Association Test (COWAT) Animal Naming North American Adult Reading Test (NAART)	COWAT—Total * Animal—Total * NAART—Total
Processing Speed	WAIS-R Digit Symbol (DS) Trail Making Test A (TMT-A) Serial Response Time (SRT) Lexical Decision Task (LDT)	DS—Total TMT-A—Total SRT—Average RT LDT—average accuracy, average RT

* Measures used for classification and therefore not employed in the dispersion computation.

## Footnotes

1 The lack of observed group differences between the AD and HC groups may be due to the presence of anosognosia in the AD individuals with respect to their memory functioning.

## Supplementary Materials

The following are available online at http://www.mdpi.com/2079-3200/6/1/12/s1, Group differences on neuropsychological tests between the health control (HC), amnestic Mild Cognitive Impairment (a-MCI) and Alzheimer’s Disease (AD) groups. Standard deviations are presented in parentheses. Post-hoc comparisons are based on Tukey’s HSD.
