# Supplementary material for: Intraindividual Variability across Neuropsychological Tests: Dispersion and Disengaged Lifestyle Increase Risk for Alzheimer’s Disease
Source: J Intell. 2018 Mar 1;6(1):12. doi: 10.3390/jintelligence6010012 (PMC6480779; doi:10.3390/jintelligence6010012)
Supplement: Supplementary file 1 [file jintelligence-06-00012-s001.pdf]

Journal of *Intelligence*

MDPI

Supplemental Material

Intraindividual Variability Across Neuropsychological Tests: Dispersion and Disengaged Lifestyle Increase Risk for Alzheimer's Disease

Drew W. R Halliday, Robert S. Stawski, Eric S. Cerino, Correne A. DeCarlo, Karl Grewal and Stuart W. S. MacDonald

Group differences on neuropsychological tests between the health control (HC), amnestic Mild Cognitive Impairment (a-MCI) and Alzheimer's Disease (AD) groups. Standard deviations are presented in parentheses. Post-hoc comparisons are based on Tukey's HSD.

	HC	a-MCI	AD
MAT 1	48.39 (7.92)	49.24 (10.71)	59.29 (11.38) *+
MAT 2	50.06 (9.70)	49.05 (10.70)	54.81 (7.23)
MAT 3	52.60 (8.82)	49.78 (9.58)	39.56 (9.62) *+
Digit Span Foreword	52.74 (10.51)	49.40 (8.62)	40.24 (3.31) *
Digit Span Backward	51.86 (9.45)	50.87 (9.27)	39.16 (7.94) *+
3MS	55.21 (3.61)	49.72 (5.45) *	28.59 (11.16) *+
NAART	54.17 (8.13)	47.84 (7.97) *	39.51 (13.16) *
Trail Making Test A	46.90 (4.03)	49.98 (4.32)	63.36 (23.74) *+
Trail Making Test B	45.41 (3.47)	48.99 (5.30) *	73.00 (8.90) *+
RAVLT Total	57.02 (6.58)	46.00 (4.52) *	33.06 (6.31) *+
RAVLT A6	57.54 (6.65)	44.09 (5.96) *	37.11 (3.23) *+
RAVLT A7	57.47 (6.53)	43.76 (6.89) *	38.51 (2.15) *
Benton VRT	54.96 (3.12)	48.73 (7.44) *	32.91 (15.70) *
Digit Symbol	54.96 (8.26)	47.95 (7.23) *	35.47 (7.73) *+
Block Design	50.85 (8.77)	51.68 (9.54) *	40.84 (12.20) *
Similarities	53.57 (5.76)	51.93 (4.17)	42.22 (15.56)
Word Fluency	54.11 (7.12)	48.11 (10.69)	39.77 (8.49) *
Animal Fluency	54.50 (8.67)	48.56 (7.34) *	35.44 (7.02) *
ALQ Total	53.94 (7.57)	48.08 (8.97)	40.01 (14.08) *

Note: * $p < 0.05$ for HC contrast; + $p < 0.05$ for a-MCI contrast.
